# An alcohol-focused intervention versus a healthy living intervention for problem drinkers identified in a general hospital setting (ADAPTA): study protocol for a randomized, controlled pilot trial

**DOI:** 10.1186/1745-6215-14-117

**Published:** 2013-04-30

**Authors:** Judith Watson, Gillian Tober, Duncan Raistrick, Noreen Mdege, Veronica Dale, Helen Crosby, Christine Godfrey, Charlie Lloyd, Paul Toner, Steve Parrott

**Affiliations:** 1 Department of Health Sciences, University of York, Heslington, York YO10 5DD, UK; 2 Leeds Addiction Unit, 19 Springfield Mount, Leeds LS2 9NG, UK

**Keywords:** Alcohol-related hospital admissions, Pilot study, Healthy living, Acceptability, AUDIT, Problem drinkers, Integrated social behavioral network therapy, Economic evaluation, Non-help seekers, Randomized controlled trial

## Abstract

**Background:**

Alcohol misuse is a major cause of premature mortality and ill health. Although there is a high prevalence of alcohol problems among patients presenting to general hospital, many of these people are not help seekers and do not engage in specialist treatment. Hospital admission is an opportunity to steer people towards specialist treatment, which can reduce health-care utilization and costs to the public sector and produce substantial individual health and social benefits. Alcohol misuse is associated with other lifestyle problems, which are amenable to intervention. It has been suggested that the development of a healthy or balanced lifestyle is potentially beneficial for reducing or abstaining from alcohol use, and relapse prevention. The aim of the study is to test whether or not the offer of a choice of health-related lifestyle interventions is more acceptable, and therefore able to engage more problem drinkers in treatment, than an alcohol-focused intervention.

**Methods/design:**

This is a pragmatic, randomized, controlled, open pilot study in a UK general hospital setting with concurrent economic evaluation and a qualitative component. Potential participants are those admitted to hospital with a diagnosis likely to be responsive to addiction interventions who score equal to or more than 16 on the Alcohol Use Disorders Identification Test (AUDIT). The main purpose of this pilot study is to evaluate the acceptability of two sorts of interventions (healthy living related versus alcohol focused) to the participants and to assess the components and processes of the design. Qualitative research will be undertaken to explore acceptability and the impact of the approach, assessment, recruitment and intervention on trial participants and non-participants. The effectiveness of the two treatments will be compared at 6 months using AUDIT scores as the primary outcome measure. There will be additional economic, qualitative and secondary outcome measurements.

**Discussion:**

Development of the study was a collaboration between academics, commissioners and clinicians in general hospital and addiction services, made possible by the Collaboration in Leadership in Applied Health Research and Care (CLAHRC) program of research. CLAHRC was a necessary vehicle for overcoming the barriers to answering an important NHS question – how better to engage problem drinkers in a hospital setting.

**Trial registration:**

ISRCTN47728072

## Background

Alcohol misuse is a major cause of premature mortality and ill health. Europe has the highest number of alcohol-related disabilities in the world with alcohol accounting for approximately 1.8 million deaths [1]. Alcohol is a contributing factor to over 60 types of disease and injury [2]. Providing specialist treatment for problem drinkers can reduce health-care utilization and costs to the public sector and result in substantial individual health and social benefits [3-5].

The number of people admitted to hospital with alcohol-related illness or alcohol misuse problems is increasing in England [6]. The majority of these people are not help seekers for their alcohol problem, nor are they identified or referred by hospital staff for specialist addiction treatment [7,8]. The hospital admission is an opportunity for identification and treatment, and the Royal College of Physicians recommends screening for alcohol problems as part of routine admission [9]. Many staff are reluctant to raise the question of problem drinking and consider this a low priority even where it is a contributory factor to the admission. Also alcohol misuse tends to be overlooked when it is not the presenting problem [10,11]. Hospital staff have expressed reluctance to use the hospital admission opportunity to intervene for a number of reasons including role legitimacy, time and pessimism about the outcome [11].

The acceptability of a treatment is one factor in engagement and retention in psychological therapies [12]. Typically, acceptability has been inferred from dropout rates, rather than being explored in its own right. Staff are more likely to undertake screening and an intervention if they believe it to be acceptable to patients [13]. Alcohol screening and treatment involve patients being asked to reveal potentially sensitive information about their drinking. Research, while somewhat contradictory, suggests that a key issue here is how such questioning is undertaken. For instance in one study, while a majority of patients reported preferring computerized feedback about their drinking (over personalized feedback from primary care staff), most patients also stated that they had no objections to being asked about their alcohol use within primary care settings [14]. This suggests that there may be multiple proxies for intervention acceptability such as the acceptability of the staff delivering the intervention (for example, rapport) [15] as well as time needed and convenience.

Marlatt and colleagues [16,17] suggest the development of a healthy or balanced lifestyle is potentially beneficial for reducing alcohol use, abstaining from alcohol use and for relapse prevention. A population-based, pre-randomized, controlled study of the effectiveness of lifestyle counseling focusing on smoking, physical activity, diet and alcohol demonstrated that a multi-factorial lifestyle approach could improve long-term alcohol habits in a general population [18]. Despite their potential, healthy lifestyle approaches have received the least emphasis in the alcohol treatment literature [19].

This study will investigate whether a healthy lifestyle approach may be more acceptable, and therefore more effective, in a non-help seeking population of problem drinkers than an explicit emphasis on changing drinking behavior alone. A standard treatment for alcohol problems is integrated social behavior and network therapy (iSBNT), which combines effective mechanisms of change in the treatment of addictive behavior, namely motivational enhancement, behavior change techniques and social support [20]. The healthy living intervention that will be compared to iSBNT has been developed on the basis of the same mechanisms with the aim of changing drinking behavior through change in one or more lifestyle domains.

## Methods/design

### Design

**Figure 1:**
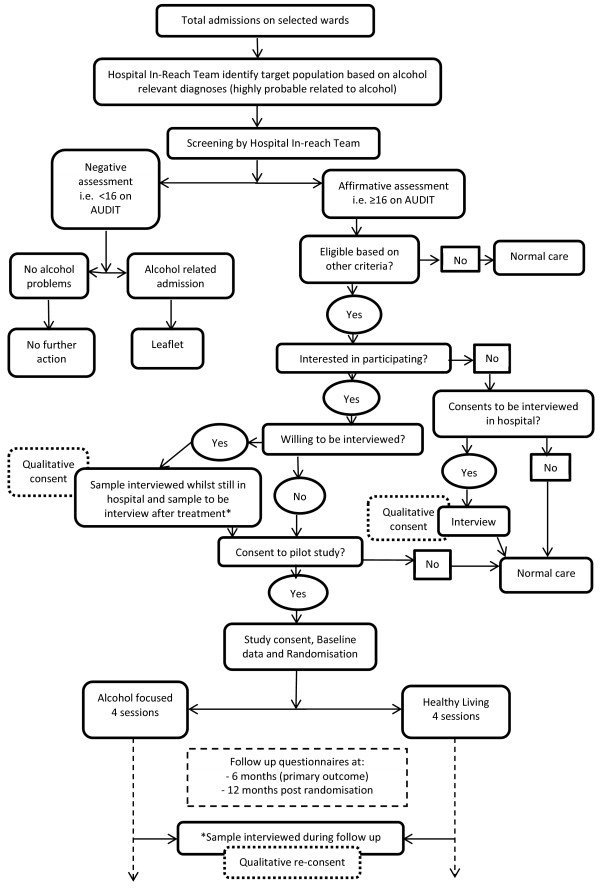
Flow diagram of ADAPTA study.

The study is a pragmatic, parallel-group, randomized, controlled pilot study in which an alcohol-focused intervention is compared with a healthy living intervention for problem drinkers identified in a general hospital setting. The study has been granted ethical approval by the National Research Ethics Service Committee Yorkshire and The Humber – Leeds Central (Reference: 11/YH/0448). A full flow diagram for the study is shown in Figure 1.

### Aims of the study

To explore through qualitative interviews, the acceptability and impact of the initial approach, assessment and attempted recruitment on participants and non-participants in the trial.

To explore through qualitative interviews, the acceptability of the healthy living intervention and the alcohol-focused intervention in a population of non-help seeking problem drinkers and alcohol-dependent patients presenting in the hospital setting.

To compare the clinical and cost-effectiveness of a healthy living intervention and an alcohol-focused intervention in their impact on drinking problems.

To compare the effects of a healthy living and alcohol-focused intervention on: quantity and frequency of drinking, dependence, social satisfaction, psychological health and health-related quality of life.

To evaluate the level of treatment retention amongst participants randomized to the two interventions, based on numbers attending first and subsequent sessions.

To explore the acceptability and feasibility of postal assessment instruments at 6- and 12-month follow-ups.

To establish patient preferences between study interventions prior to treatment.

### Hypothesis

The hypothesis is that the healthy living intervention with its broader focus on up to three lifestyle domains out of a choice of diet, exercise, smoking, drinking, personal care, drug use and medication concordance will have greater acceptability and therefore be more effective than a more specific focus on a change of alcohol drinking behavior in a non-help seeking population of problem drinkers.

### Participants and setting

The study is being undertaken in two general hospitals where alcohol-related admissions have been increasing year on year [21]. Preparatory studies in our program of research used a two-stage process to identify wards with a) the highest rates of admissions of patients with alcohol-related diagnoses and b) where there would be benefit from treating the alcohol problem. These wards are the main but not exclusive source of recruitment of patients into the study.

Staff from the local NHS addiction treatment unit currently provide an in-reach service to these hospitals, which aims to engage patients in specialist treatment and effect an early hospital discharge. The in-reach team are responsible for recruiting and providing treatment to the study participants. All patients admitted to the target wards are considered potential participants if they meet the eligibility criteria.

### Eligibility criteria

Patients are eligible if they:

– are admitted to one of the hospitals with a diagnosis thought likely to be responsive to addiction interventions

– score equal to or more than 16 on the Alcohol Use Disorders Identification Test (AUDIT)

– are aged 18 years and over (males and females)

– are willing and able to give written informed consent

Patients are excluded if they:

– have received specialist treatment with a primary focus on alcohol in the past 6 weeks

– have no fixed abode (that is, are not available for follow-up)

– are currently serving a sentence in prison or have outstanding legal issues likely to lead to imprisonment

– have a mental or physical illness likely to preclude active participation in treatment or follow-up (for example, are not stable through current medication or treatment)

– are unwilling or unable to give written informed consent

– are unable to take part in either intervention using spoken English or are unable to self-complete the validated English language outcome measure tools

### Study procedures

#### Identification

All patients admitted to the selected wards are considered potential participants. The hospital in-reach team identifies patients who have an alcohol-related diagnosis or a reason for admission that suggests a possible alcohol-related diagnosis, which is thought to be responsive to addiction interventions.

#### Eligibility assessment

Eligibility is assessed using a screening form, which includes the AUDIT plus a checklist for the other eligibility (inclusion/exclusion) criteria. Patients with an AUDIT score ≥16 who meet the remaining eligibility criteria are provided with a patient information sheet (PIS). All patients with an AUDIT score ≥16, but who do not meet the remaining eligibility criteria continue with their care as current practice dictates.

Patients scoring 15 or less on the AUDIT who have been admitted with an alcohol-related problem are given a leaflet containing information on units, stopping or reducing alcohol intake, the support available at a local specialist service, the address of an interactive website designed to guide people towards reducing drinking and are offered treatment as usual. The leaflet is based on Department of Health advice and evidence from previous trials including the Screening and Intervention Programme for Sensible Drinking trial [22].

Completed screening forms, including anonymous ones for those not eligible or not willing to enter the trial, are returned to the coordinating center. This information is used to collate eligibility and non-eligibility or non-participation information, as well as providing AUDIT scores, reason for admission, age and gender for prevalence data and to inform future studies.

#### Consent procedure

Potential participants are provided with a verbal explanation of the study and a copy of the PIS. The PIS outlines the purpose of the study, the proposed interventions, study processes and time commitment required for both treatment and follow-up assessments. For those agreeing to participate, written consent is obtained and a baseline questionnaire is completed. An additional PIS gives information and asks for consent to participate in the qualitative element of the study.

#### Randomization process

Six therapists are divided into three pairs, broadly matched on experience and qualifications. Within each pair, one therapist is trained to deliver the alcohol-focused intervention and the other trained to deliver the healthy living treatment. Each participant is randomized such that they have a 50:50 chance of getting the therapist who screened them or the matched colleague. This approach is based on previously obtained opinions from service users stating that continuity of care is important to them. Should one of the therapists in the pair not be available due to illness or similar reasons, the participant is allocated to a therapist within a different pair at the point of randomization. Participants are randomized by a secure remote computer service run by the fully registered York Trials Unit, to either an alcohol-focused intervention (control treatment) or a healthy living intervention.

#### Intervention content

Both interventions are delivered in four sessions using motivational dialogue based on the guiding principles of motivational interviewing [23]. This number of sessions has been determined using evidence from the UK Alcohol Treatment Trial (UKATT), which suggest optimal attendance is achieved over four sessions [24].

Each intervention consists of four sessions of 30 to 45 minutes each, delivered one to two weeks apart, over a maximum period of 8 weeks. Sessions for either arm can be delivered at the specialist clinic, the participant’s home or at a mutually agreed suitable alternative location as is current practice for the Hospital In-Reach Team (HIRT).

Both interventions involve prompting the formation of intention through encouraging participants to set a general goal or make a behavioral resolution, and encouraging them to set specific goals, self-monitor behavior using a diary. Feedback from the participant on their performance allows them to review the behavioral goals together with the therapist. Motivational dialogue is used to elicit behavior change by helping clients explore and resolve ambivalence and facilitate treatment goal-setting [20].

The alcohol-focused intervention (iSBNT) is a social-network-based, cognitive behavioral intervention using motivational dialogue [20] and drawing on cognitive behavioral therapy (CBT), which has been shown to be an effective approach for treating patients with alcohol problems [25-27]. It has a strong evidence base in UK clinical trials and is increasingly commonly taught and practiced in the UK addiction field. It contains exclusively drinking behavior change topics and involves strengthening coping skills and confidence, with emphasis on structure and skill rehearsal. Behavioral rehearsal is used incrementally to develop the service user’s and network members’ skill levels and confidence to apply new skills in their own environment. Homework is used to implement new practice and facilitate achievement of agreed treatment goals.

The participant is encouraged to recruit a supportive network of concerned others, who will help the participant to implement behavior change and develop new lifestyle habits inconsistent with drinking. The network is encouraged to make plans to cope with relapse risks and actual relapse, providing support for each other where necessary.

The healthy living intervention, which is also a behavioral approach, combines behavior change in up to three chosen domains that contribute to overall health. Seven options from which the participant chooses three are diet, exercise, drinking, smoking, medication concordance, drug use and personal care. Choice is a key variable in the study as it is the basis of exploring acceptability [28]. The participant is encouraged to enlist the help of someone who is concerned about them and broadly shares their behavioral goals. At the first session, the target domains are agreed. These are not set in stone but are likely to be the ones to be pursued. The participant is then asked in which order they wish to pursue the domains and a goal is set for the first one. Behavioral plans are made for achieving the goal and homework is set to put these plans into practice. At the second session, progress towards the previously set goal is explored and reviewed and the next domain is addressed in the same way. This continues in the third and fourth sessions. Where the behavioral goals for a domain are not achieved, the therapist and participant decide whether to repeat or to move on to the next domain. The participant is encouraged to identify and bring along a buddy to support the intervention and this can be the same person or separate individuals for each domain. Drinking does not have to be one of the chosen domains.

Concurrent treatment can occur wherever there is a need for medical treatment of a physical condition, or surgical or psychiatric treatment that does not preclude delivery of the study interventions. If after their participation in the research is complete, a participant wishes to continue with specialist addiction treatment, they can request this and their referral will be treated in the normal way.

#### Training

Six addiction practitioners working in the hospital in-reach team received dedicated training covering the study’s procedures and the practices and techniques of each intervention, followed by supervised practice of study treatments until deemed competent. The training package is delivered separately to the two treatment groups by an experienced trainer/addiction psychologist. Once deemed competent, practitioners continue to deliver the manually guided study interventions under regular supervision based on recorded practice to avoid therapist drift. Additional training is given on the study protocol process, documentation completion and obtaining informed consent.

#### Blinding

Due to the nature of the interventions in this study, blinding of participants and therapists to allocated treatment is not possible. However, baseline data were collected prior to randomization and follow-up data will be collected by postal questionnaires: those involved in the analysis of the data are blind to treatment allocation.

#### Qualitative data

Although assessing the components and processes of the design is an important feature of this pilot, the main purpose is to evaluate the acceptability of the interventions to the participants. Participant interviews are being undertaken to explore the acceptability and impact of approach, assessment, recruitment and intervention on trial participants and non-participants (those not willing to take part in the trial, but do consent to an interview). A purposive sample of 20 to 30 eligible participants are being interviewed face-to-face while still in hospital – both those who have expressed an interest in taking part in the trial and non-participants, exploring their thoughts and feelings concerning their identification as a problem drinker in the hospital context and how this status compares to their own perceptions of their drinking behavior. This also allows us to explore the impact of hospitalization and related health concerns on motivation to engage in treatment. The interviews with the non-participants also probe reasons for not participating in the study.

Semi-structured interviews (mixture of face-to-face and telephone) are also being undertaken with a sample of 10 to 15 individuals from each arm of the trial who have participated in part or all of their treatment sessions. These interviews examine a broad range of issues including their responses to the baseline assessment, how the social engagement functions (integral to both interventions) have worked, the perceived obstacles to change and how formal elements of the interventions have interacted with other aspects of their lives, including significant life events and changes of circumstances. The data from these interviews will add to the quantitative measures in addressing the acceptability of these interventions and the possible mechanism of their impacts. Importantly, they ensure that patient perspectives are included in the evaluation of these interventions.

In addition, the therapists delivering the interventions will be interviewed once recruitment is well under way to ask them about their experience of identifying people with alcohol issues and how patients responded to their approach. The therapists will be provided with an information sheet and written consent will be obtained.

All interviews are being conducted by an experienced mixed-methods researcher using the appropriate topic guide/ schedule for the sample being interviewed (that is, in hospital, post treatment or therapist) to promote consistency. Where permitted, interviews are audio-recorded. In addition, a second researcher will independently perform a thematic analysis on a sub-sample of transcripts, with discussions taking place to develop an agreed coding framework.

#### Patient outcome measures

This study also provides an opportunity to compare the effectiveness of the two interventions. Therefore, the AUDIT score at 6 months post randomization has been designated as the main patient outcome and is also measured at 12 months post randomization, whilst the proportion of participants who have not consumed any alcohol in the previous 6 months is collected via a yes/no question at 6 and 12 months post randomization.

A number of secondary patient outcomes are also measured. The Leeds Dependence Questionnaire (LDQ) [29], a ten-item, self-administered measure designed to assess severity of dependence is helpful in determining a patient’s treatment goals [30]. Social satisfaction is measured using the Social Satisfaction Questionnaire (SSQ), a validated eight-item scale found to be a suitable outcome measure in people with substance use disorders covering social domains that are likely to be important to, and engage, service users in treatment [31].

Subjective well-being, psychological problems and functioning are measured using the Clinical Outcomes in Routine Evaluation (CORE-10) [32]. The health-related quality of life (HRQoL) is assessed using the European Quality of Life – 5 Dimensions (EQ-5D). The EQ-5D is a standardized measure of health status developed by the EuroQol Group in order to provide a simple, generic measure of health for clinical and economic appraisal, where health is characterized on five dimensions (mobility, self-care, ability to undertake usual activities, pain and anxiety/depression) [33]. These four measures are taken at baseline, and 6 and 12 months post randomization.

At the end of the second and third treatment sessions, both participant and therapist are asked to complete the 12-item (short version) working alliance inventory (WAI) [34] in order to assess the participant–therapist relationship.

The costs of delivering the two interventions are calculated on the basis of resources used. Participant use of health services, other alcohol services outside the study, public services and criminal justice services is assessed through service use-questions at baseline, and 6 and 12 months post randomization. We will use utility values based on societal values using the York tariff [35].

Recruitment rates are presented in three tiers: number screened, number eligible and number participating, with the last of these used to infer initial acceptability. Retention in treatment is evaluated by the number of sessions attended and used as a measure of treatment acceptability. Quantitative assessment of the acceptability of the postal follow-up questionnaires is assessed by return rates and completion rates. Qualitative assessment of any issues regarding questionnaire completion is explored during the interviews. Patients’ reported preferences for either or neither of the study interventions are collected in the baseline questionnaire, prior to randomization.

All follow-up data are collected by postal questionnaire at 6 and 12 months post randomization with postal reminders sent 2 and 4 weeks after initial correspondence. In the event of a participant failing to complete their questionnaires, attempts to obtain at least the primary outcome data are made via telephone.

#### Quality assurance of treatment delivery

All treatment sessions are video-recorded (the participant is not visible) for the purpose of supervision in order to prevent practitioner drift. These are independently evaluated to rate the delivery of the essential and unique components of each treatment using an instrument adapted from the UKATT process rating scale [36].

### Recruitment

Recruitment was originally planned to continue for 8 months. However, due to an unexpected delay in the commencement of recruitment, combined with the additional factor of a hard end date for recruitment, this was reduced to 5 months.

#### Sample size calculation

Originally the trial had planned to use 6 therapists over 8 months. It was estimated from current case loads that 4 therapists can screen a maximum of 384 patients in 6 months. Of these, 113 (29.4%) would engage with the specialist service. The maximum number screened over 8 months was estimated at 768 or 96 per month. Using the number interested in being treated as an estimate of those who would consent to the trial, the maximum number we could have recruited was approximately 222 (that is, 111 in each group) which is 28 per month. Based on experience in previous studies where loss to follow-up has been in the region of 20%, we erred on the side of caution, allowing for a follow-up rate of 70%. This should have resulted in approximately 77 patients in each group. With 77 patients in each group, using 80% power, two-sided 5% significance, we would have been able to detect an effect size difference of 0.45 on the AUDIT score at month 6.

However, because the recruitment period has been reduced to 5 months, the maximum number that can be screened is 480, of which we could potentially recruit 140. Using the same follow-up rates, power and significance as before, this would enable an effect size difference of 0.57 on the AUDIT score at month 6 to be detected.

### Analysis

The main aims of this pilot study are to evaluate the acceptability of the interventions to the participants, and the elements and processes of the design. Using the opportunity available to compare the effectiveness of the two interventions and advising whether one or both could be taken forward into a large-scale study, the planned analyses cover all aspects.

#### Qualitative analysis

Interviews are digitally recorded (where consent is given), fully transcribed and imported into Atlas.ti [37] for the management and analysis of data. Thematic analysis will be used to identify themes by their frequency, intensity and extensiveness, a technique described by Braun and Clarke [38].

#### Primary statistical analysis

The primary patient outcome measure for the study is the AUDIT score measured at 6 months. Analysis will be on an intention-to-treat basis using two-sided, 5% significance and will compare outcomes for the alcohol-focused intervention with outcomes for the healthy living intervention at 6 months.

The AUDIT scores at 6 months will be analyzed using a linear regression model. The baseline AUDIT score will be included as a covariate and the model will also include the treatment group. Model checking will be performed to ensure that the model is a good fit for the data. If necessary, transformations will be used to improve model fit.

#### Secondary statistical analyses

Secondary outcomes include the AUDIT score at month 12. They also include LDQ, SSQ, CORE-10 and EQ-5D all measured at 6 and 12 months. These will be analyzed separately for each time point using linear regression models, adjusted for the baseline score of the dependent variable and include a variable for treatment group. All analyses will compare the alcohol-focused intervention with the healthy living intervention.

Abstinence, a binary outcome (yes/no) collected at month 6 and month 12, will be analyzed using logistic regression models for each time point. Included in the models will be a variable for treatment group and baseline AUDIT score.

Preference for treatment will be collected prior to randomization and will be summarized overall and by treatment group. The primary analysis will be repeated but with the addition of the variable for treatment preference and a variable for the interaction between preference and treatment group to examine the effect preference may have on outcomes.

The relationship between client and therapist as measured by the WAI will be explored using descriptive statistics and correlation where appropriate. We will undertake an exploratory analysis to investigate the effect of adherence to treatment using a complier average causal effect analysis [39,40]. The numbers of adherent participants and further details of adherence will also be summarized.

#### Economic analysis

The economic component of the study comprises a cost-effectiveness and cost-utility analysis, and aims to identify, quantify and value health-care resources utilized by participants in each trial arm.

Resources utilized in the identification and delivery of the alcohol-focused and healthy living interventions will be recorded. This will allow the calculation of costs related to the implementation of these interventions. Local costs will be used to calculate the costs of the interventions. In addition, specific training costs for staff are calculated in terms of staff time, costs for premises and the cost of training materials.

Participants’ use of health care will be identified retrospectively using service use questions and applying a common set of national unit cost estimates [41,42]. Participant costs in the 6-month period before the intervention can then be compared to costs in the 6-month period after receiving the intervention and used to explore any changes in costs imposed by participants in each group.

The economic analysis will calculate the incremental cost-effectiveness of the alcohol-focused intervention compared to the healthy living intervention, using measures of clinical outcome and quality of life (EQ-5D [33]) responses at baseline and at 6 and 12 month follow-up. The use of EQ-5D enables the estimation of quality-adjusted life years (QALYs). Data will be bootstrapped to account for the expected skewness evident in economic cost data. The analysis will construct cost-effectiveness acceptability curves to illustrate the probability that the alcohol-focused intervention is more cost-effective than healthy living, based on different monetary values being attached to the QALYs. The use of QALYs follows the recommendations of the National Institute for Health and Clinical Excellence [43] and enables the value for money afforded by treatment to be compared to a range of other health-care interventions.

#### Treatment fidelity analyses

Visual recording of all treatment sessions is used to rate the quantity and quality of the treatment components delivered. This rating is based upon an instrument adapted from the UKATT process rating scale [36] and performed by a researcher trained in process rating. Correlational analysis of the data derived from rating is performed to detect protocol adherence and discriminability between the treatments.

## Discussion

### The CLAHRC context

The NIHR-funded Collaboration for Leadership in Applied Health Research and Care (CLAHRC) created the opportunity for this pilot study; the ethos of CLAHRC is collaboration and the implementation of research findings, which is well demonstrated in this work. A number of preparatory studies based on collaborations between the general hospital trust, two universities, a specialist NHS clinic and commissioners of alcohol services were necessary steps in forming the background and context of the design and delivery of the pilot. These studies enabled the identification of where the pilot would be conducted, namely the target wards; who the pilot would target, namely the diagnostic categories; what the interventions would be, the nature of current practice and therefore extrapolations of recruitment rates and the practicalities of procedures. Systematic reviews of screening, assessment instruments and interventions for problem drinkers informed the choice of methods used. Running in parallel, CLAHRC also provided the resources for delivering a training program to all clinical care givers in the two general hospitals, which was designed to enhance attitudes and practice in identifying problem drinkers and initiating a conversation that would lead to a referral to a specialist service. The CLAHRC collaboration agreed on the importance of the research questions and was an enabling group that drove the program forward.

### The challenges for the pilot study

The CLAHRC training project addressed the challenge of getting hospital staff on board. The scope of the training, in covering all the target wards, was made possible by the collaboration with senior managers. CLAHRC was a vehicle for improving the existing shared working between hospital staff and the specialist in-reach service and in taking steps towards the pilot study; improvements in practice were achieved, for example, the identification of problem drinkers on the wards, and the accessibility and acceptance of the specialist team.

### The benefits of the pilot study

The specialist hospital in-reach team staff are enhancing their repertoire of specialist skills by adding an intervention that can be offered, where appropriate and preferred, in treatment as usual. They are given regular training and supervision in this intervention alongside existing practice.

This study will improve our understanding of the acceptability and impact of these interventions on lifestyles and drinking, and will inform future research questions regarding this key target group. The findings will be used to shape the design of a multi-center trial with this patient population and setting.

## Trial status

At the time of submission, participants are actively being recruited into this study and a number of qualitative interviews are being conducted.

## Abbreviations

ADAPTA: Addressing Drinking Among Patients comparing Two Approaches; ARiAS: Alcohol Research in Acute Settings; AUDIT: Alcohol Use Disorders Identification Test; CBT: cognitive behavioral therapy; CLAHRC: Collaboration for Leadership in Applied Health Research and Care; CORE-10: Clinical Outcomes in Routine Evaluation 10 items; EQ-5D: European Quality of Life 5 Dimensions; HIRT: Hospital In-Reach Team; iSBNT: integrated social behavioral and network therapy; LDQ: Leeds Dependence Questionnaire; NIHR: National Institute for Health Research; PIS: patient information sheet; QALY: quality-adjusted life year; SSQ: social satisfaction questionnaire; UKATT: UK Alcohol Treatment Trial; WAI: working alliance inventory.

## Competing interests

The authors declare that they have no competing interests.

## Authors’ contributions

The ARiAS research team had the original idea for the study and all authors contributed to the final design. CG, DR and GT secured the CLAHRC funding for the ARiAS theme, which includes this pilot study. GT and DR are responsible for the clinical management of the study. CL and CG are responsible for the academic research aspects of the study, to which GT and DR contribute. HC is responsible for study co-ordination and overseeing data collection at the clinical site. JW has overall responsibility for the study management supported by NM at the coordinating center. VD and SP are responsible for the statistical and health economic design respectively. PT is responsible for the design, conduct and analysis of the qualitative work. JW wrote the first draft of the study protocol and all the authors contributed to the editing. All authors read and approved the final manuscript.
